# Perceptions of the Role of Living Alone in Providing Services to Patients With Cognitive Impairment

**DOI:** 10.1001/jamanetworkopen.2023.29913

**Published:** 2023-08-18

**Authors:** Elena Portacolone, Tung T. Nguyen, Barbara J. Bowers, Julene K. Johnson, Ashwin A. Kotwal, Robyn I. Stone, Sahru Keiser, Thi Tran, Elizabeth Rivera, Paula Martinez, Yulin Yang, Jacqueline M. Torres, Kenneth E. Covinsky

**Affiliations:** 1 Institute for Health & Aging, University of California, San Francisco; 2 Phillip R. Lee Institute for Health Policy Studies, University of California, San Francisco; 3 Division of General Internal Medicine, School of Medicine, University of California, San Francisco; 4 School of Nursing, University of Wisconsin, Madison; 5 Division of Geriatrics, Department of Medicine, University of California, San Francisco; 6 LeadingAge, Washington, DC; 7 Department of Epidemiology and Biostatistics, University of California, San Francisco

## Abstract

**Importance:**

The potential role of living alone in either facilitating or hampering access to and use of services for older adults with cognitive impairment is largely unknown. Specifically, it is critical to understand directly from health care and social services professionals how living alone creates barriers to the access and use of supportive health care and social services for racially and ethnically diverse patients with cognitive impairment.

**Objective:**

To identify the potential role of living alone in the access and use of health care and social services for diverse patients with cognitive impairment by investigating professionals’ perceptions of caring for such patients who live alone in comparison with counterparts living with others.

**Design, Setting, and Participants:**

This qualitative study of 76 clinicians, social workers, and other professionals used semistructured interviews conducted between February 8, 2021, and June 8, 2022, with purposively sampled professionals providing services to diverse patients with cognitive impairment in Michigan, California, and Texas.

**Main Outcomes and Measures:**

Clinicians, social workers, and other professionals compared serving patients with cognitive impairment and living alone vs counterparts living with others. An inductive content analysis was used to analyze the interview transcripts.

**Results:**

A total of 76 professionals were interviewed (mean [SD] age, 49.3 [12.7] years); 59 were female (77.6%), 8 were Black or African American (11%), and 35 were White (46%). Participants included physicians, nurses, social workers, and home-care aides, for a total of 20 professions. Participants elucidated specific factors that made serving older adults living alone with cognitive impairment more challenging than serving counterparts living with others (eg, lacking an advocate, incomplete medical history, requiring difficult interventions), as well as factors associated with increased concerns when caring for older adults living alone with cognitive impairment, such as isolation and a crisis-dominated health care system. Participants also identified reasons for systematic unmet needs of older adults living alone with cognitive impairment for essential health care and social services, including policies limiting access and use to public home-care aides.

**Conclusions and Relevance:**

In this qualitative study of professionals’ perspectives, findings suggest that living alone is a social determinant of health among patients with cognitive impairment owing to substantial barriers in access to services. Results raised considerable concerns about safety because the US health care system is not well equipped to address the unique needs of older adults living alone with cognitive impairment.

## Key Points

**Question:**

How do health care and social services professionals perceive the role of living arrangements when they care for patients with cognitive impairment?

**Findings:**

In this qualitative study, 76 professionals elucidated specific factors that made serving older adults living alone with cognitive impairment more challenging than serving counterparts living with others. Factors included isolation, a crisis-dominated health care system, and policies limiting access to public home-care aides.

**Meaning:**

Findings suggest that living arrangements are a social determinant of health among patients with cognitive impairment because those living alone are more likely to experience gaps in services and they are harder to serve than counterparts living with others.

## Teaser

This qualitative study assesses clinician and social worker perceptions of the role of living alone in providing services for patients with cognitive impairment.

## Introduction

In the United States, it is estimated that 4.3 million older adults (≥55 years) live alone with cognitive impairment (ranging from mild cognitive impairment to dementia),^1^ which represents 25% of older adults living with cognitive impairment.^1^ These individuals are likely to be female, poorer, widowed, or divorced and to have never been married.^1^ Older adults living alone with cognitive impairment are at an elevated risk for untreated medical conditions,^2,3,4^ self-neglect,^3,5,6,7^ malnutrition,^6,8^ and falls.^5,6,8^ Evidence suggests that these negative health outcomes are associated with a lack of long-term services and supports from family members, friends, or formal caregivers (eg, home-care aides).^9,10,11,12,13,14,15,16,17^

Older adults living alone with cognitive impairment often have limited access to long-term services and supports because they have limited access to unpaid informal supports from spouses and children,^1,18,19,20,21^ who provide most of the long-term services and supports to people with cognitive impairment in the United States.^12^ Furthermore, only an estimated 21% of older adults living alone with cognitive impairment are covered by Medicaid,^1^ which leaves most of this population ineligible for publicly subsidized home-care aides and other services covered by Medicaid.^1^ Thus, older adults living alone with cognitive impairment in the United States tend to have difficulty consistently accessing essential health care and social services, which constitutes a public health problem, yet little is known about the perspectives of health care and social services professionals who have expertise caring for this population.

Understanding the potential role of living alone in accessing essential long-term services and supports can inform the development of programs and policies to ensure access to these services for older adults living alone with cognitive impairment, especially racial and ethnic minority groups, who have increased risk of cognitive impairment vs White individuals,^22,23,24,25^ often experience poor access to long-term services and supports,^26,27,28,29,30^ and often live alone.^31,32^ To identify specific mechanisms by which living alone might be associated with the provision of care to patients with cognitive impairment, we interviewed a wide range of health care and social services professionals serving diverse patients with cognitive impairment.

## Methods

### Study Design

The design of this qualitative study (ie, type of professionals and questions) was reviewed by the Policy Advisory Group and the Community Advisory Board of the Living Alone with Cognitive Impairment Project.^33^ Qualitative research methods were used because they are appropriate to expand knowledge on unexplored areas in which hypothesis generation is premature. To foster an exploratory perspective reflecting the multiple needs of older adults with cognitive impairment, we recruited professionals from different specialties. Professionals were recruited from organizations mostly in California and Michigan. Some professionals were recruited via referrals of our Policy Advisory Group, Community Advisory Board, and research team, and via snowballing, which led to recruiting professionals in Texas. Other professionals were identified via organizations’ websites and recruited via email or telephone calls. The review board at the University of California, San Francisco approved the study. The Standards for Reporting Qualitative Research (SRQR) reporting guidelines were followed.^34^

### Interviews

Sociologists with PhD training in advanced qualitative methods (E.P. and B.J.B.) designed the semistructured interview guide, drawing from 3 pilot interviews. This study is a portion of a larger qualitative study on barriers or facilitators to access to services for older adults living alone with cognitive impairment, with attention to race and ethnicity.^33^ Interviews were conducted over the telephone or via video from February 8, 2021, to June 8, 2022, after participants gave verbal informed consent and, in an electronic survey, self-reported their demographic characteristics and the demographic characteristics in which they serve. Researchers (E.P., E.R., and P.M.) first asked, “How is it like to care for people with cognitive impairment?” If the role of living alone emerged with this question, we probed (eg, “Could you please tell me more”). If the role of living alone did not emerge, we then asked, “Is there any difference between caring for patients with cognitive impairment living alone, as opposed to those living with others?” To elicit the richest data, we asked for specific examples. To ensure reflexivity, after each interview researchers recorded in field notes their reactions to what was observed during interviews and their potential role.^34,35^

### Statistical Analysis

Transcripts of audiotaped interviews and field notes were uploaded into ATLAS.ti, version 9 (Scientific Software Development GmbH). We used inductive content analysis^36^ to understand the potential role played by living alone in providing services to patients with cognitive impairment. For example, participants often explained that older adults living alone with cognitive impairment are more “challenging” to serve than counterparts living with others. As a result, sentences with such statements were coded “older adults living alone with cognitive impairment, + challenging.” Initially, transcripts were analyzed line by line by 1 researcher (E.P.) to identify the potential role played by living arrangements in facilitating or hampering access or use of services for people with cognitive impairment. A code was created every time a particular comparison was identified and its description detailed in the coding manual. Next, interpretative convergence was achieved by 1 researcher (E.P.) and the senior coder (T.T.) through researcher explanation of the coding criteria and by iterative review of the senior coder’s coded transcripts until the criteria were mirrored.^37^ Next, the senior coder trained 2 researchers, who coded the rest of the transcripts. Interpretative convergence was achieved after both coders mirrored the senior coder’s criteria, which occurred after independent coding of 4 transcripts.^37^ Afterward, the senior coder reviewed each coded transcript; thus, each transcript was coded by 2 researchers. Furthermore, the researcher (E.P.) regularly reviewed the coded transcripts.^37^ Additional codes were added with the approval of the research team.^32^ After all transcripts were coded, saturated themes were identified by one of the researchers (E.P.) by making connections among codes, writing memos, and having iterative discussions with the team.^37,38^ A large sample of interviews facilitated saturation.^38,39^ Data were analyzed from June 1, 2021, to August 10, 2022.

## Results

Seventy-six health care and social services professionals (mean [SD] age, 49.3 [12.7] years) were interviewed. A total of 17 were male (22%), 59 were female (78%), 21 were Asian (28%), 8 were Black or African American (11%), 5 were Hispanic or Latinx (7%), and 35 were White (46%). As Table 1 illustrates, professionals included physicians, nurses, social workers, and home-care aides, representing a total of 20 professions. Most professionals cared for patients with cognitive impairment who resided in urban areas and were racially and ethnically diverse. Interviews lasted approximately 1 hour. Professionals elucidated factors that made serving older adults living alone with cognitive impairment particularly challenging, as well as factors associated with increased concerns about safety when serving older adults living alone with cognitive impairment. Narratives further identified reasons for the systematic unmet needs for essential services for this population. Representative quotes are displayed in Table 2.

**Table 1.** Characteristics of Health Care and Social Services Professionals

Characteristic	No. (%)
Age, mean (SD), y^a^	49.3 (12.7)
Sex	
Male	17 (22)
Female	59 (78)
Race and ethnicity (professionals able to select all that applied)	
American Indian or Alaska Native	0
Asian	21 (28)
Black or African American	8 (11)
Hispanic or Latinx	5 (7)
Native Hawaiian or Pacific Islander	0
White	35 (46)
>1 Race	7 (9)
Other	0
Current job title	
Home-care services administrator	10 (13)
House services coordinator	8 (11)
Caseworker	7 (9)
Home-care aide	7 (9)
Physician	7 (9)
Nurse	7 (9)
Social worker	7 (9)
Adult day care center administrator	6 (8)
Long-term care insurance agent	3 (4)
Elder-law attorney	2 (3)
Health care organization administrator	2 (3)
Psychiatrist	2 (3)
Adult Protective Services manager	1 (1)
Information technology officer	1 (1)
Director of aging programs	1 (1)
Meal services administrator	1 (1)
Neurologist	1 (1)
Occupational therapist	1 (1)
Pharmacist	1 (1)
Reverend	1 (1)
Language spoken during interview	
English	71 (93)
Cantonese or Mandarin	4 (5)
Spanish	1 (1)
Communities served, urban or rural (professionals able to select all that applied)	
Rural	6 (8)
Urban	59 (78)
Rural and urban	11 (14)
Communities served, race and ethnicity (professionals able to select all that applied)^b^	
American Indian or Alaska Native	26 (34)
Asian	58 (76)
Black or African American	58 (76)
Hispanic or Latinx	57 (75)
Native Hawaiian or Pacific Islander	31 (41)
White	58 (76)
Other^c^	3 (4)
State	
California	43 (57)
Michigan	31 (41)
Texas	2 (3)

^a^ Available for 72 participants (4 missing).

^b^ “Please describe the communities that you or your organization serves.”

^c^ Responses included Arab American, Middle Eastern, and Russian.

**Table 2.** Quotes From Health Care and Social Services Professionals

Theme and subtheme	Quote
Factors that make serving OALACI more challenging	Super challenging [to serve OALACI]. Because … sometimes you have to find things like meals, like just basic, how are they going to live? Are they safe? Are they just forgetting a couple of little things or are they forgetting like turning off their stove? Can they really still live by themselves? … Who’s doing your medication? (Nurse)
Lacking advocate	The challenge for me is trying to access services for him or her … if they don’t have family. So that’s the hardest part, because then we have to get the community involved, and kind of coordinating, that can sometimes take an act of God. (APS manager)
Incomplete medical history	The other thing you’re missing is history. You may think the person’s declining but you have no idea at what point they were declining or what they were like before then, unless there’s a family member involved. (Pharmacist)
“Falling off the radar”	Are they [OALACI] able or not able to really take care of themselves? That’s a difficult discussion, especially when they start missing appointments and we don’t necessarily have the staff to really try to reach out to them. And the main way that we reach out to people is primarily through phone calls, and people who are older or have cognitive disabilities may not necessarily respond to an unknown phone call. (Physician)
“Painstaking” intervention	Sometimes I have to call APS 2 or 3 times to get them involved in a case. And unfortunately sometimes that person has to get really, really bad before services are actually put in for that resident, because if that resident has any type of … capacity to make some decisions for themselves, then APS sometimes won’t always get involved. And when that person was assigned, let’s say a guardian ad litem or whatever, I found that that wasn’t helpful either because … just trying to find good … external support for a resident, I found it could be quite difficult. (House service coordinator)
Factors associated with increased concerns about safety when serving OALACI	I wish I could say we were able to make arrangements and they [OALACI] could go and live somewhere that’s safe for them, but like I said, sometimes it takes months to find the right place, and then it’s just us going in there and doing patchwork repair to make sure that they stay safe. Because we can’t always say, “Well, do you have a daughter or son or a sister or whomever that can take care of you?” (Nurse)
OALACI isolation deflects formal supports	Folks [living alone with cognitive impairment] know that they’re not doing well, but they are so scared of losing their independence and concerned about people coming into their homes and seeing that they may be struggling, that they may decline [our services] out of fear. (Director of aging programs [state agency])
Tension between ensuring safety vs honoring values	A lot of our patients don’t like the threat to their independence.… And so they’re worried and suspicious that somebody’s going to come in and take their autonomy away from them. So you have to deal with a lot of those fears too, which in many cases they’re right. (Nurse)
Crisis-dominated system	We see a whole bunch of … emergency calls come in like right at Thanksgiving or at Christmas.… The adult child comes home for the holidays and looks around and goes, something’s wrong here. Mom’s forgetting things. Mom’s not keeping the house clean like she used to.… So we suddenly get a bunch of panicked calls: what do I do? Where do I go? (Adult day care center administrator)
Reasons for OALACI systematic unmet needs for essential professional services	If in-home care could be more affordable or more easily accessible, or you didn’t have to either have a ton of money or be really, really low income to qualify, then people living with cognitive impairment could live alone and could be—get the support that they need and could stay in their home for longer, if that’s what they wish. (Social worker)
OALACI narrow social networks	So most of them [patients with cognitive impairment] live alone, and they have no one listed as emergency contact, no one, not a family member, not even a friend to rely on in case of a crisis. (Case manager)
OALACI less likely to visit clinics and offices	To go to the doctor sometimes is not a fun thing for them [referring to his client living alone with cognitive impairment]. They want to stay at home where they’re comfortable, where they can kind of control the environment…. Sometimes the appointments are scheduled so far out they forget why I’m even having this appointment. Why am I seeing this doctor? Why am I having this appointment? (Home-care aide)
Policies hampering access to long-term services and supports	We force people who make a dollar too much to have to go to the nursing home to access Medicaid benefits for long-term care, as opposed to being cared for somewhere in the community…. So that can be a real challenge … because we can’t get people cared for where they most want to be cared for, unless they just have the money to pay for it. So that can be difficult. (Elder-law attorney)

Abbreviations: APS, Adult Protective Services; OALACI, older adults living alone with cognitive impairment.

### Factors That Make Serving Older Adults Living Alone With Cognitive Impairment More Challenging

Most participants explained that serving older adults living alone with cognitive impairment was an “ongoing battle” requiring considerably more effort than serving counterparts living with others. Specifically, participants elucidated 4 factors that made serving older adults living alone with cognitive impairment more challenging.

#### Lacking an Advocate

Older adults living alone with cognitive impairment often lacked an advocate or “copilot” supporting them to enact participants’ recommendations regarding medications, diet, and precautions. As a result, serving these older adults often required increased effort from participants, including repeating recommendations and reminders. Despite these efforts, encounters often ended without participants’ knowing whether recommendations would be followed. Language barriers exacerbated these challenges. These extra efforts, compounded with the uncertainty about adherence, affected participants’ distress. Conversely, when reflecting on patients living alone or with others with advocates, participants pointed to the burden placed on caregivers.

#### Incomplete Medical History

Participants often had difficulty acquiring a comprehensive medical history of older adults living alone with cognitive impairment, whereas counterparts living with others typically had someone who could offer collateral information. Therefore, it was more difficult for participants to assess the specific needs of older adults living alone with cognitive impairment.

#### “Falling Off the Radar”

Older adults living alone with cognitive impairment were more likely to miss visits because they either forgot or preferred avoiding professionals. Serving these older adults via telehealth was often difficult owing to trouble navigating video conferencing without technical supports. Driving to visit these older adults in rural areas was also sometimes financially disadvantageous to professionals because of compensation schemes. Protocols in some memory clinics requiring companions further affected the difficulty of regularly visiting older adults living alone with cognitive impairment.

#### “Painstaking” Intervention

A prominent challenge was how to intervene once participants concluded that older adults living alone with cognitive impairment no longer had the capacity and supports to continue living by themselves. A combination of 3 factors complicated this “painstaking process.” First, whereas some professionals had positive experiences when involving Adult Protective Services, others found that calling had limited effects. Limitations of Adult Protective Services included not taking a case because it was deemed not alarming enough, reporting that patients were “fine” or “sound of mind,” and recommending limited in-home interventions. Second, when professionals initiated the process to assign a public guardian to older adults living alone with cognitive impairment, this conservatorship process took months because of waiting lists, thereby protracting concerns about patients’ safety. Some professionals also explained that public guardians may not be fully invested in supporting their patient because these officers often oversaw numerous clients in precarious circumstances. Third, these patients often resisted the decision of conservatorship and distanced themselves from professionals.

### Factors Associated With Increased Concerns About Safety When Serving Older Adults Living Alone With Cognitive Impairment

Most participants spontaneously expressed their concerns about the safety of older adults living alone with cognitive impairment because of their exposure to risks. “Who’s watching them?” one Adult Protective Services manager asked. “Nobody,” he replied. Participants’ concerns about safety included “not eating,” “not taking care of personal hygiene,” “leaving gas stove on,” “mixing up their medications,” “wandering off,” falling, “driving,” and financial abuse. Sentinels of unsafe cases were missed appointments, rotten food in the refrigerator, weight loss, “mail piling up,” and “dirty” clothes. Participants pointed to 3 factors that made their safety concerns difficult to manage.

#### Isolation Deflecting Supports

The isolation of some older adults living alone with cognitive impairment was associated with increased participant concerns. Isolation stemmed from combinations of living alone, having limited informal networks, having cognitive impairment that hampered the ability of negotiating social activities, residing in underserved neighborhoods (eg, rural, high crime), and avoiding interferences. Furthermore, a tendency to keep personal matters private affected distancing from professionals for this group. Interviewees often assumed that the isolation of some older adults was due to distrust in professionals. Isolation also stemmed from fear of “forcible removals from their home if they are not managing well.” As a nurse reflected: “Somebody who really doesn’t want anybody involved, really likes their privacy, really sees our suggestions to get more help as a threat, and they are not able to identify anyone…[t]hat’s…the riskiest case.” Professionals often distinguished between older adults living alone with cognitive impairment who were receptive to their recommendations vs those difficult to work with because of unresponsiveness to recommendations, being difficult to reach, or not allowing people into their homes.

#### Tension Between Ensuring Safety and Honoring Autonomy

A tension emerged between professionals’ need to ensure safety while respecting preferences for self-determination and independence of older adults living alone with cognitive impairment. Cultural mores affected the tension. For example, professionals explained that their safety interventions were pitted against the high value that their patients or clients placed on protecting their privacy, as well as continuing to cook, drive, and live in their home.

#### Crisis-Dominated System

Operating within a fragmented health care system with limited supports for older adults living alone with cognitive impairment fueled concerns about safety. One consequence of this limited infrastructure was that these patients were sometimes identified in emergency departments after crises (eg, medication mismanagement, falls) and were often discharged without support systems in place. For example, on a hospital discharge in which an older adult was given taxi vouchers to return home, a psychiatrist reflected that it was “like sending a kid out to play on the freeway.” Participants’ concerns also increased when patients and clients living alone with cognitive impairment resided in underserved and high-crime neighborhoods in either rural or urban counties. Regarding the discharge of a patient living alone with cognitive impairment in Detroit, a nurse recalled, “She had to take a bus to get back, and then still walk another couple blocks to a house that was ransacked while she was in the hospital.”

### Reasons for Systematic Unmet Needs for Essential Services for Older Adults Living Alone With Cognitive Impairment

Participants’ narratives elucidated reasons for experiencing unmet needs for professional services for older adults living alone with cognitive impairment. Critical services included having help with taking medications, keeping up with payments and “paperwork,” navigating telephone calls with professionals (eg, utilities), having transportation to medical appointments, cleaning the home, buying groceries, and having help with dressing and laundry. Compared with counterparts living with others, older adults living alone with cognitive impairment needed more frequent and extended “checks” for professionals to assess the situation. Reasons ranged from narrow social networks to federal reimbursement policies.

#### Narrow Social Networks

The living-alone status often pointed to narrow social networks and limited presence of family members, who are the foremost supports of people with cognitive impairment in the United States. Professionals often noted their relief if the patient with cognitive impairment lived with someone. Reasons for limited supports from families included widowhood, childlessness, adult children’s competing obligations, discords among family members, and families being “spread out across the nation” or, for immigrants, across the globe. Because of the limited unpaid supports from family members and friends, older adults living alone with cognitive impairment had greater needs for professional services than counterparts living with others. Owing to the salience of unpaid supports for persons with cognitive impairment in the United States, professionals often spontaneously differentiated between older adults living alone with cognitive impairment who relied on some family support and those who did not, stressing that those without any supports were particularly at risk for adverse health outcomes.

#### Older Adults Living Alone With Cognitive Impairment Less Likely to Visit Clinics and Offices

Despite extensive need for formal services for older adults living alone with cognitive impairment, professionals working in clinics (eg, physician and nurses) or offices (eg, elder-law attorneys) noted that “typically” they were visited by persons who were accompanied by caregivers who lived with them. Family members often advocated and navigated the services on behalf of the person with cognitive impairment. It was less common to have older adults living alone with cognitive impairment visit their offices. Conversely, it was more common to treat these older adults in hospital emergency departments postcrises, such as falls.

#### Policies Hampering Access to Long-Term Services and Supports

To prevent crises, health care and social services professionals often stressed the importance for older adults living alone with cognitive impairment to have extra “eyes” and “ears” via home-care aides, as well as nurses and physicians who made home visits. The interviewed home-care aides underscored how important it was for them to support their clients living alone. A few home-care aides worried about not knowing what would happen after they ended their day shift. When reflecting on the care of older adults or clients living alone with cognitive impairment, professionals often pointed to the issue of the limited access to home-care aides. On one hand, only a portion of these older adults (ie, those receiving Medicaid) could access publicly funded home-care aides for limited hours. For example, one publicly funded home-care aide explained that she was “authorized” to spend a maximum of 6 hours daily with her client living alone with cognitive impairment; as soon as she observed that her client required extra hours, she had to report that the client might need “placement.” On the other hand, most patients who live alone are ineligible for Medicaid. As a result, they or their families, if available, must pay “thousands and thousands of dollars” out of pocket to access adequate care to either continue living in their home by hiring private home-care aides or relocate into an assisted living facility, which were practices observed with some affluent patients. Because affording these private home and community-based services in the long term was often prohibitive and informal unpaid supports were unavailable, older adults living alone with cognitive impairment were prone not to receive adequate care. As one caseworker noted, “I can’t pull a support system out of nowhere, especially without money.”

## Discussion

This qualitative study illuminated professionals’ perspectives about offering health care and social services to diverse patients with cognitive impairment and living alone in the United States. Participants identified challenges to provision of care, access to essential services, and substantial safety concerns. Together, participants’ insights suggested that older adults living alone with cognitive impairment are systematically destined to fall through the cracks of a health care system overly relying on unpaid caregivers, limited access to public home-care aides, and a reactionary, crisis-driven system. These social forces may be associated with the demonstrated negative health consequences observed for older adults living alone with cognitive impairment.^2,3,4,5,6,7,8,40^

Participants spontaneously identified that older adults living alone with cognitive impairment who had strained relationships with professionals and no informal networks were at particularly heightened risk. These findings matter because they pointed to specific modifiable mechanisms that hamper access to services to a population in extreme need of professional services owing to their age, cognitive impairment, and narrow social networks.

This study adds to efforts to improve the care, services, and supports of persons with cognitive impairment and their caregivers by highlighting specific challenges associated with living alone. Although robust literature has highlighted the importance of addressing the burden for unpaid caregivers to care for someone with cognitive impairment,^12,41^ the novelty of this study was in emphasizing the burden of disease on professionals caring for clients with cognitive impairment who live alone, their living-alone status a marker of uncertainties and narrow social networks.

This investigation highlighted the specific challenges of serving patients with cognitive impairment and living alone. This study expanded findings of a Canadian investigation^42^ that also highlighted the tension between supporting older adults living alone with cognitive impairment while preserving their independence. Similarly, professionals’ concern to ensure the safety of older adults living alone with cognitive impairment emerged in both studies, which indicated that ensuring safety is an essential component of supports because older adults in this group are prone to adverse events.^2,3,4,5,6,8^ Our study expanded this Canadian investigation of 15 participants (mostly nurses) by focusing on the specific role of living arrangements and including a wider range of health care and social services professionals.

This study drew from health care and social services professionals’ perspectives to illuminate the structural mechanisms leading to systematic deficiencies in adequately responding to the basic needs of older adults living alone with cognitive impairment. A key structural mechanism is a health care system not designed to prevent crises among this group, as well as not providing appropriate supports. Evidence of this flawed design is that older adults living alone at diagnosis of cognitive impairment, or even after hospitalizations, do not receive bolstered supports.^18^ Professionals, on the other hand, have limited directions and resources on how to intervene when their patients with cognitive impairment lack capacity and adequate supports to continue to live alone. Public policies exacerbate this flawed design by allowing only a fraction of older adults living alone with cognitive impairment in the United States—we estimated 21%^1^—to qualify to receive essential home-care aides via Medicaid.

In accordance with these findings, we call for studies testing interventions and proposing policy solutions addressing these structural factors. For example, identifying older adults living alone with cognitive impairment who are at heightened risk (eg, those without nearby and invested caregivers) is imperative. Next, designing specific formal supports (eg, dedicated case managers, home-care aides, medical home visits) for these older adults and identifying resources (eg, expansion of Medicare and Medicaid) to fund these extra supports is also critical. The eligibility for Medicaid coverage based on health and functional status varies by state, and this flexibility may allow for an expansion of services to more older adults living alone with cognitive impairment.

Addressing the unique needs of older adults living alone with cognitive impairment should be a priority of the National Alzheimer’s Project Act requiring the secretary of the Department of Health and Human Services to provide adequate supports to people with cognitive impairment.^43,44^ We suggest creating an interagency workgroup focused on older adults living alone with cognitive impairment, bringing together agencies with shared responsibilities to this population (eg, Administration for Community Living, Centers for Medicare & Medicaid Services). International comparisons of best practices serving older adults living alone with cognitive impairment might also inform US-based interventions. For example, in Denmark older adults in this group are identified and followed up by local authorities who routinely offer home-based supports.^21^

Findings suggested that living arrangements are a social determinant of health among patients with cognitive impairment because such patients who live alone are more likely to experience gaps in services because they are more challenging to serve than counterparts living with others. Consequently, within comprehensive assessments of social determinants of health, the living-alone status of patients with cognitive impairment should be automatically flagged, given specific challenges facing health care and social services professionals in serving those patients. A detailed understanding of whether living arrangements hamper access to and use of care is important because evidence of systemic gaps in services and lower quality of care between comparable yet distinct groups (eg, older adults living alone with cognitive impairment vs counterparts living with others) may ultimately suggest that the most vulnerable group might be prone to health disparities.^45^ More studies are needed to understand how older adults living alone with cognitive impairment experience systemic gaps in services and lower quality of care than counterparts living with others.

### Limitations

Limitations of this study include recruiting health care and social services professionals active in 3 states, with only 2 professionals active in Texas. Furthermore, sampling represented a range of professions but was too broad (1 of each of several professions) to allow for a detailed understanding of professional-specific issues. In addition, our analysis did not examine heterogeneity in experiences across race and ethnicity, sexual orientation, gender identity, and other factors among patients; future studies should focus on heterogeneity across these groups. Furthermore, professionals were not offered the option to review the interview transcripts for additional feedback and approval. Finally, a standard limitation of qualitative research is limited generalizability of findings, which can be addressed by replicating this study in other states and drawing from quantitative methods.^46^

## Conclusions

In this qualitative study of health care and social services professionals’ perspectives, findings underscored the need for interventions and policies to support older adults living alone with cognitive impairment. Such interventions and policies will become increasingly critical because effective treatments to reverse the course of cognitive impairment are unavailable, childlessness and divorce are common,^47,48^ and older adults are projected to live longer and often alone.

## Notes

Supplement.
Data Sharing Statement
