# Supplementary material for: Perceptions of the Role of Living Alone in Providing Services to Patients With Cognitive Impairment
Source: JAMA Netw Open. 2023 Aug 18;6(8):e2329913. doi: 10.1001/jamanetworkopen.2023.29913 (PMC10439475; doi:10.1001/jamanetworkopen.2023.29913)
Supplement: Supplement. — Data Sharing Statement [file jamanetwopen-e2329913-s001.pdf]

Data Sharing Statement

Data
Data available: Yes
Data types: Deidentified participant data, Data (not involving human participants)
How to access data: Request for data must be sent to an individual, please email elena.portacolone@ucsf.edu
When available: beginning date: 01-01-2024

Supporting Documents
Document types: Informed consent form
How to access documents: Request for data must be sent to an individual, please email elena.portacolone@ucsf.edu
When available: beginning date: 01-01-2024

Additional Information
Who can access the data: researchers whose proposed use of the data has been approved
Types of analyses: To support better public policies and programs to support older adults
Mechanisms of data availability: Data will be available with a signed data access agreement
